# Effect of sitagliptin on energy metabolism and brown adipose tissue in overweight individuals with prediabetes: a randomised placebo-controlled trial

**DOI:** 10.1007/s00125-018-4716-x

**Published:** 2018-08-25

**Authors:** Kimberly J. Nahon, Fleur Doornink, Maaike E. Straat, Kani Botani, Borja Martinez-Tellez, Gustavo Abreu-Vieira, Jan B. van Klinken, Gardi J. Voortman, Edith C. H. Friesema, Jonatan R. Ruiz, Floris H. P. van Velden, Lioe-Fee de Geus-Oei, Frits Smit, Lenka M. Pereira Arias-Bouda, Jimmy F. P. Berbée, Ingrid M. Jazet, Mariëtte R. Boon, Patrick C. N. Rensen

**Affiliations:** 1 Division of Endocrinology, Department of Medicine, Leiden University Medical Center, post zone C7Q, P. O. Box 9600, 2300 RC Leiden, the Netherlands; 2 Einthoven Laboratory for Experimental Vascular Medicine, Leiden University Medical Center, Leiden, the Netherlands; 3 PROFITH ‘Promoting Fitness and Health through Physical Activity’ research group, Department of Physical Education and Sport, Faculty of Sport Sciences, University of Granada, Granada, Spain; 4 Department of Human Genetics, Leiden University Medical Center, Leiden, the Netherlands; 5 Division of Vascular Medicine, Department of Internal Medicine, Erasmus Medical Center, Rotterdam, the Netherlands; 6 Division of Nuclear Medicine, Department of Radiology, Leiden University Medical Center, Leiden, the Netherlands; 7 Department of Nuclear Medicine, Alrijne Hospital, Leiderdorp, the Netherlands

**Keywords:** Brown adipose tissue, Diabetes risk, DPP4 inhibitor, Dyslipidaemia, Energy expenditure, Obesity, Prediabetes, Skeletal muscle

## Abstract

**Aims/hypothesis:**

The aim of this study was to evaluate the effect of sitagliptin on glucose tolerance, plasma lipids, energy expenditure and metabolism of brown adipose tissue (BAT), white adipose tissue (WAT) and skeletal muscle in overweight individuals with prediabetes (impaired glucose tolerance and/or impaired fasting glucose).

**Methods:**

We performed a randomised, double-blinded, placebo-controlled trial in 30 overweight, Europid men (age 45.9 ± 6.2 years; BMI 28.8 ± 2.3 kg/m^2^) with prediabetes in the Leiden University Medical Center and the Alrijne Hospital between March 2015 and September 2016. Participants were initially randomly allocated to receive sitagliptin (100 mg/day) (*n* = 15) or placebo (*n* = 15) for 12 weeks, using a randomisation list that was set up by an unblinded pharmacist. All people involved in the study as well as participants were blinded to group assignment. Two participants withdrew from the study prior to completion (both in the sitagliptin group) and were subsequently replaced with two new participants that were allocated to the same treatment. Before and after treatment, fasting venous blood samples and skeletal muscle biopsies were obtained, OGTT was performed and body composition, resting energy expenditure and [^18^F] fluorodeoxyglucose ([^18^F]FDG) uptake by metabolic tissues were assessed. The primary study endpoint was the effect of sitagliptin on BAT volume and activity.

**Results:**

One participant from the sitagliptin group was excluded from analysis, due to a distribution error, leaving 29 participants for further analysis. Sitagliptin, but not placebo, lowered glucose excursion (−40%; *p* < 0.003) during OGTT, accompanied by an improved insulinogenic index (+38%; *p* < 0.003) and oral disposition index (+44%; *p* < 0.003). In addition, sitagliptin lowered serum concentrations of triacylglycerol (−29%) and very large (−46%), large (−35%) and medium-sized (−24%) VLDL particles (all *p* < 0.05). Body weight, body composition and energy expenditure did not change. In skeletal muscle, sitagliptin increased mRNA expression of *PGC1β* (also known as *PPARGC1B*) (+117%; *p* < 0.05), a main controller of mitochondrial oxidative energy metabolism. Although the primary endpoint of change in BAT volume and activity was not met, sitagliptin increased [^18^F] FDG uptake in subcutaneous WAT (sWAT; +53%; *p* < 0.05). Reported side effects were mild and transient and not necessarily related to the treatment.

**Conclusions/interpretation:**

Twelve weeks of sitagliptin in overweight, Europid men with prediabetes improves glucose tolerance and lipid metabolism, as related to increased [^18^F] FDG uptake by sWAT, rather than BAT, and upregulation of the mitochondrial gene *PGC1β* in skeletal muscle. Studies on the effect of sitagliptin on preventing or delaying the progression of prediabetes into type 2 diabetes are warranted.

**Trial registration:**

ClinicalTrials.gov NCT02294084.

**Funding:**

This study was funded by Merck Sharp & Dohme Corp, Dutch Heart Foundation, Dutch Diabetes Research Foundation, Ministry of Economic Affairs and the University of Granada.

**Electronic supplementary material:**

The online version of this article (10.1007/s00125-018-4716-x) contains peer-reviewed but unedited supplementary material, which is available to authorised users.

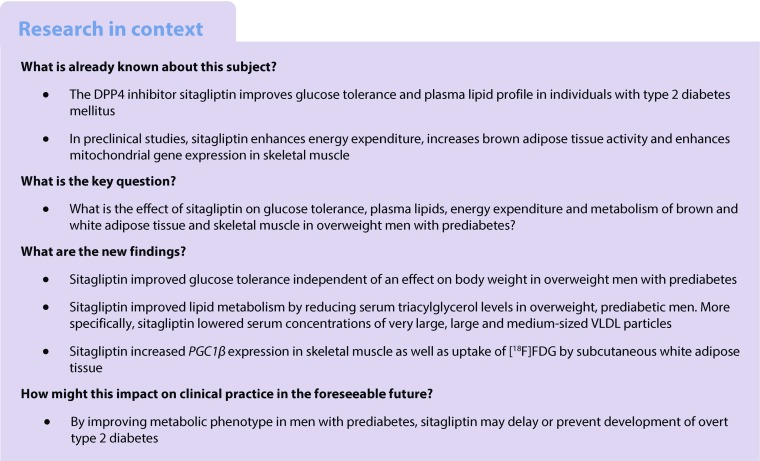



## Introduction

The worldwide prevalence of obesity is rapidly increasing [1]. In obese individuals, glucose homeostasis is frequently dysregulated, leading to a condition known as prediabetes (impaired glucose tolerance and/or impaired fasting glucose). It is estimated that up to 70% of individuals with prediabetes eventually develop type 2 diabetes mellitus [2]. Therefore, delaying or preventing the development of type 2 diabetes is a potentially fruitful therapeutic strategy. In individuals with type 2 diabetes, glucose dysregulation is often accompanied by atherogenic dyslipidaemia, characterised by high triacylglycerol and LDL-cholesterol levels and low HDL-cholesterol levels [3, 4]. Current treatment strategies are aimed at targeting both hyperglycaemia and dyslipidaemia by combining glucose-lowering drugs with statins. However, a drug that improves both glucose and lipid profile might be of specific interest.

The dipeptidyl peptidase-4 (DPP4) inhibitor sitagliptin, which enhances the bioavailability of incretin hormones, improves both glucose tolerance and lipid metabolism in individuals with type 2 diabetes [5–7], thereby targeting both microvascular and macrovascular complications. The precise mechanism by which sitagliptin exerts these positive metabolic effects remains largely unknown. Interestingly, preclinical data with sitagliptin point towards a mechanism that involves the enhancement of energy expenditure through an increase in the activity of energy-combusting brown adipose tissue (BAT) and/or enhanced skeletal muscle respiratory capacity [8].

BAT combusts glucose and fatty acids towards heat, thereby increasing energy expenditure [9]. High amounts of BAT, at least judged from the ability of BAT to take up [^18^F] fluorodeoxyglucose ([^18^F]FDG), are associated with lower plasma glucose levels and higher insulin sensitivity in humans [10–12] and BAT prevalence is inversely correlated with diabetic status [13]. In addition, recruitment of BAT by short-term exposure to cold alleviates peripheral insulin resistance in individuals with type 2 diabetes [14]. Of note, mouse studies have shown that BAT activation can also improve lipid profile and attenuate atherosclerosis development [15]. Notably, in white adipose tissue (WAT) depots [16] inducible ‘beige’ adipocytes are present that have thermogenic capacity and can contribute to energy expenditure (reviewed in [17]).

Human skeletal muscle is an important contributor to energy expenditure and whole-body glucose metabolism. Insulin resistance of skeletal muscle is one of the earliest abnormalities that precede type 2 diabetes, and mitochondrial dysfunction has been implicated in the underlying pathogenesis [18]. Improving human skeletal muscle metabolism, including mitochondrial function, is therefore another important therapeutic goal to ameliorate insulin resistance.

Although many studies have focused on improving the metabolic profile of individuals with type 2 diabetes, less attention has been paid to identifying therapies that improve glucose and lipid levels in prediabetic individuals. This may be of particular benefit since most of these individuals eventually proceed to overt diabetes [2, 19]. Therefore, the aim of this study was to evaluate the effect of sitagliptin on glucose tolerance, plasma lipids, energy expenditure, skeletal muscle metabolism and uptake of [^18^F] FDG by BAT and WAT in overweight individuals with prediabetes.

## Methods

For extensive descriptions of the methods used, see the electronic supplementary material (ESM) Methods.

### Participants

Thirty-two overweight (BMI 25–35 kg/m^2^) Dutch Europid men with prediabetes, aged 35–55 years, were recruited. Prediabetes was defined as having a fasted serum glucose between 5.6 mmol/l and 6.9 mmol/l, according to the ADA criteria [20], and/or a plasma glucose level between 7.8 mmol/l and 11.1 mmol/l following an OGTT, according to WHO criteria for impaired glucose tolerance. Exclusion criteria were type 2 diabetes (e.g. fasted glucose >6.9 mmol/l and/or plasma glucose following OGTT >11.1 mmol/l), smoking, recent weight change, rigorous exercise, uncontrolled chronic disease or a positron emission tomography/computed tomography (PET/CT) scan within the last year. The study was approved by the Medical Ethical Committee of the Leiden University Medical Center (LUMC) and performed in accordance with the principles of the revised Declaration of Helsinki. Written informed consent was obtained from all volunteers prior to participation. Thirty participants were initially randomised. Two participants withdrew from the study prior to completion (both in the sitagliptin group–one participant because of heartburn and one participant because of joint pain) and were subsequently replaced with two new participants that were allocated to the same treatment (see ESM Fig. 1). Thus, 30 participants completed the study. After completion of the study, one participant from the sitagliptin group was excluded from analyses due to a distribution error where the participant received both sitagliptin and placebo as treatment.

### Study design

Participants were enrolled in a randomised, double-blinded, placebo-controlled study and received oral administration of either sitagliptin (100 mg/day sitagliptin phosphate; Januvia; Merck Sharp and Dome, Haarlem, the Netherlands) or placebo for 12 weeks. The primary study endpoint was the effect of sitagliptin on BAT volume and activity. Secondary endpoints were the effects of sitagliptin on body weight and composition, resting energy expenditure (REE), glucose tolerance, fasting markers in blood for glucose and lipid metabolism, [^18^F] FDG uptake by WAT and skeletal muscle and gene expression in skeletal muscle biopsies. As a post hoc analysis, we quantified lipid and lipoprotein composition using high-throughput proton nuclear magnetic resonance (NMR) metabolomics. Participants were studied at baseline and after 12 weeks of treatment. Before and after the treatment period, two measurement days took place. During the first day, body composition was measured (DEXA; iDXA; GE Healthcare, Little Chalfont, UK), followed by an individualised water-cooling protocol as described previously [21] (see ESM Methods). Thermoneutral and cold-exposed fasting blood samples were obtained and REE was measured by indirect calorimetry (Oxycon Pro; CareFusion, Heidelberg, Germany) (see ESM Methods) and skin temperature was measured (iButton; Maxim Integrated Products, San Jose, CA, USA). In addition, cold-induced [^18^F] FDG uptake by BAT, WAT and skeletal muscle was determined by PET/CT scan (Gemini TF-64; Philips Healthcare, Best, the Netherlands) 1 h after administration of 110 MBq of [^18^F] FDG (see ESM Methods). In the sitagliptin group, one participant became claustrophobic inside the PET/CT scan and could not finish this measurement. On the second day, a fasted skeletal muscle biopsy was taken from the vastus lateralis muscle [22] and then an OGTT was performed (see ESM Methods). All measurements took place after participants had fasted for 10 h overnight and had consumed a standardised dinner the night before. Each week during the treatment period, participants measured their blood glucose and were contacted to monitor compliance and adverse events.

### Serum measurements

Commercially available enzymatic kits were used to measure serum concentrations of triacylglycerol and total cholesterol (Roche Diagnostics, Woerden, the Netherlands), NEFA (Wako Chemicals, Neuss, Germany) and glucose (Instruchemie, Delfzijl, the Netherlands). Insulin concentrations were measured using ELISA (Crystal Chem, Elk Grove Village, IL, USA). Plasma catecholamines were measured in the laboratory of Vascular Medicine (Erasmus MC, Rotterdam, the Netherlands) using standard procedure. Aspartate aminotransferase, alanine aminotransferase, γ-glutamyltransferase, HbA_1c_ and HDL-cholesterol were determined by the general hospital Laboratory of the LUMC and LDL-cholesterol was calculated using the Friedewald equation [23]. Lipid and lipoprotein composition was quantified using high-throughput proton NMR metabolomics (Nightingale Health, Helsinki, Finland), as described previously [24]. The following components were quantified: phospholipids, triacylglycerol, total cholesterol, non-esterified cholesterol and cholesteryl esters. The mean size for VLDL, LDL and HDL particles was calculated by weighting the corresponding subclass diameters with their particle concentrations [25]. For the analysis of the OGTT, the AUC was calculated using the trapezoidal rule [26]. Incremental AUC was calculated by deducting the area below the baseline value from total AUCs. Insulin sensitivity was estimated using the Matsuda index [27]. The insulinogenic index (IGI; Δ*I*_*0–30*_*/*Δ*G*_*0–30*_, where I is insulin and G is glucose) was used as a measure of early insulin secretion [28]. The oral disposition index (DI_o_; [Δ*I*_*0–30*_*/*Δ*G*_*0–30*_]/fasting insulin) was used to estimate beta cell function relative to the prevailing level of insulin resistance [29].

### PET/CT scan analysis

[^18^F] FDG uptake by BAT, WAT and skeletal muscle was determined from the [^18^F] FDG PET/CT scan using Fiji ImageJ 1.51d (Beth, Israel) [30] and analysed by two researchers blinded to allocation. For BAT, a personalised standardised uptake value (SUV) threshold with a tissue radiodensity between −190 and −10 Hounsfield units was used [31]. For WAT, skeletal muscle and reference tissues, an SUV threshold of 0 was used and no Hounsfield units threshold was applied.

### qPCR analysis in skeletal muscle biopsies

Skeletal muscle biopsies were analysed for expression of genes involved in insulin signalling (*INSR*, *IRS1*), glucose metabolism (*GLUT4*), lipid metabolism (*ACACA*, *ACACB*, *ACSL1*, *CD36*, *FASN*) and mitochondrial function (*CTP1α*, *CTP1β*, *CTP2*, *CYCS*, *DNM1L*, *MFN2*, *OPA1*, *PPARGC1α* [also known as *PPARGC1A*], *PPARGC1β* [also known as *PPARGC1B*], *UCP3*), as well as DPP4 (*DPP4*) and fibroblast growth factor (*FGF21*), using quantitative (q) PCR (Bio-Rad CFX96; Veenendaal, the Netherlands) (see ESM Table 1 for primer sequences). Bio-Rad software version 3.1 (Bio-Rad Laboratories, Hercules, CA, USA) was used for analysis and quantification. Biopsy material from five participants (two from placebo and three from sitagliptin group) was insufficient for reliable mRNA analysis. Expression levels were normalised using the housekeeping gene β-actin (*ACTB*) and expressed as fold change using the ${2}^{-{\Delta \Delta \mathrm{C}}_{\mathrm{t}}}$ method.

### Statistical analysis

Power calculations were made for the primary outcome measurement of BAT activity (SUVmean; see ESM Methods). On the basis of previous studies [32], we anticipated a 20% increase in BAT activity after sitagliptin treatment. Assuming a bilateral alternative, we were able to detect differences of at least 20% in SUVmean, with a power of more than 80% and an *α* of 0.05 in a group of 30 participants. Data were analysed using SPSS Statistics (version 23.0; IBM Corporation, Armonk, NY, USA). Data are shown as mean ± SEM, unless stated otherwise. Two-tailed unpaired Student’s *t* test was used to compare baseline characteristics between sitagliptin and placebo group. Mixed model analyses with treatment and occasion as fixed effects and subject-specific deviances from the mean as random effects were used to assess the effect of the treatment. If the mixed model failed to converge, a non-parametric paired test (Wilcoxon signed-rank test) was used. Statistical results are shown with adjustment for multiple testing. Bonferroni-corrected levels of significance are shown in the table footnotes and figure legends.

## Results

### Participant characteristics and compliance

Thirty overweight, Europid men with prediabetes completed the study, although one participant from the sitagliptin group was excluded from analyses due to a distribution error (the participant received both sitagliptin and placebo as treatment). Characteristics of the remaining participants are summarised in Table 1. All measured baseline characteristics were comparable between the two groups (Table 1), except for alanine aminotransferase, which was higher in the placebo group (0.73 ± 0.08 vs 0.47 ± 0.05 μkat/l; *p* = 0.009 [not significant with a Bonferroni-corrected level of significance of 0.003]). A total of 83% of the participants had isolated impaired fasting glucose and 17% of the participants had combined impaired fasting glucose and impaired glucose tolerance. The daily oral administration of 100 mg sitagliptin was well tolerated and reported side effects (e.g. nasopharyngitis, heartburn and joint pains) were mild and transient and not necessarily related to the treatment. No episodes of hypoglycaemia were observed. Compliance was confirmed by counting returned tablets. In the placebo group only transient nasopharyngitis was reported.

**Table 1:** Participant characteristics at screening

Characteristic	Placebo (*n* = 15)	Sitagliptin (*n* = 14)
Age, years	47.1 (1.5)	45.3 (1.7)
Height, m	1.83 (0.02)	1.79 (0.02)
Weight, kg	93.7 (1.6)	95.4 (3.1)
BMI, kg/m^2^	27.8 (0.4)	29.6 (0.9)
Waist circumference, cm	101 (2)	100 (2)
Hip circumference, cm	101 (2)	103 (2)
Waist-to-hip ratio	1.00 (0.01)	0.98 (0.01)
Fasting glucose, mmol/l	5.9 (0.1)	5.8 (0.1)
Fasting insulin, pmol/l	81.3 (24.3)	54.2 (9.7)
HbA_1c_, mmol/l	36.5 (0.8)	36.2 (0.9)
HbA_1c_, %	5.5	5.5
Fasting triacylglycerol, mmol/l	1.70 (0.31)	1.47 (0.19)
Fasting total cholesterol, mmol/l	5.32 (0.32)	5.84 (0.24)
Aspartate aminotransferase, μkat/l	0.52 (0.03)	0.42 (0.03)
Alanine aminotransferase, μkat/l	0.73 (0.08)	0.47 (0.05)*
γ-Glutamyltransferase, μkat/l	0.62 (0.08)	0.53 (0.07)

Values are presented as mean (SEM). Data are from all participants that completed the study, apart from *n* = 1 in the sitagliptin group, who was excluded due to pharmacy error

Two-tailed unpaired Student’s *t* test was used for statistical comparison

*p =0.009 for sitagliptin vs placebo (not significant with Bonferroni-corrected level of significance of 0.003 (*α* = 0.05/16)

### Sitagliptin improves glucose tolerance without changing body weight or composition

**Fig. 1:**
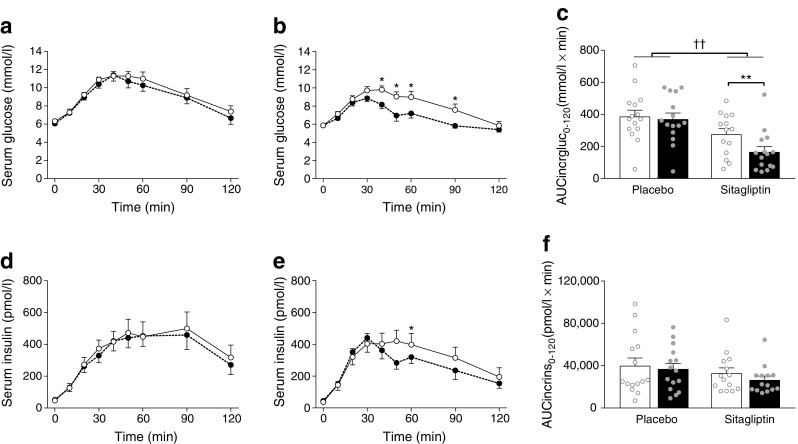
The effect of sitagliptin on glucose tolerance in overweight men with prediabetes. An OGTT was performed to assess glucose tolerance, before (white circles and bars) and after (black circles and bars) 12 weeks of placebo (*n* = 15) or sitagliptin (*n* = 14) treatment. Serum was collected before and at several time points up to 120 min after ingestion of 75 g of glucose. Glucose and insulin excursions were determined at the indicated time points after treatment with placebo (**a**, **d**) or sitagliptin (**b**, **e**). Incremental AUC (AUCincr) was calculated for glucose (gluc; **c**) and insulin (ins; **f**). Data are presented as means ± SEM. Mixed model analysis was used for statistical comparison. In (**a**, **b**, **d**, **e**) *0.006 < *p* < 0.05 for week 0 vs week 12, not significant with Bonferroni-corrected level of significance 0.006 (*α* = 0.05/9). In (**c**, **f**) **p < 0.01 for week 0 vs week 12, significant with Bonferroni-corrected level of significance 0.01 (*α* = 0.05/4); ^††^*p* < 0.01 for placebo vs sitagliptin, significant with Bonferroni-corrected level of significance 0.01 (*α* = 0.05/4)

We first assessed the effects of sitagliptin on glucose metabolism. Sitagliptin did not change fasting serum glucose or insulin levels (Table 2). However, compared with baseline, 12 weeks of sitagliptin treatment improved glucose tolerance as demonstrated by reduced glucose excursions (AUCgluc_0–120_ −15%, *p* < 0.003; Table 2), lower incremental glucose excursions (AUCincrgluc_0–120_ −40%, *p* < 0.003; Table 2; Fig. 1b, c) and lower peak glucose levels (−12%, *p* < 0.003; Table 2) during OGTT. Incremental insulin excursions (AUCincrins_0–120_) were not significantly reduced upon sitagliptin treatment (Table 2; Fig. 1e, f). Furthermore, sitagliptin improved IGI (+38%, *p* < 0.003; Table 2) and increased DI_o_ (+44%, *p* < 0.003; Table 2). Placebo did not affect either glucose or insulin excursions (Table 2 and Fig. 1a, c, d, f). Using a mixed model, we confirmed that the sitagliptin-induced changes in peak glucose levels (*p* < 0.05), AUCgluc_0–120_ (*p* < 0.05), AUCincrgluc_0–120_ (*p* < 0.003), IGI (*p* < 0.05) and DI_o_ (*p* < 0.003) were significantly different from placebo. Sitagliptin did not affect body composition (ESM Table 2), nor did it affect proximal, distal or supraclavicular skin temperature upon cooling (ESM Table 3). In addition, the liver enzymes aspartate aminotransferase, alanine aminotransferase and γ-glutamyltransferase were not affected by sitagliptin treatment (ESM Table 4). Of note, placebo increased alanine aminotransferase (+82%, *p* < 0.01; ESM Table 4).

**Table 2:** Effect of sitagliptin on measures of glucose tolerance in overweight men with prediabetes

Variable	Placebo (*n* = 15)		Sitagliptin (*n* = 14)	
	Week 0	Week 12	Week 0	Week 12
Fasting glucose, mmol/l	6.3 (0.2)	6.1 (0.2)	5.9 (0.2)	5.9 (0.2)
Peak glucose, mmol/l	12.2 (0.4)	12.1 (0.5)	11.0 (0.4)	9.7 (0.3)**^,†^
Peak glucose time, min	52.7 (4.8)	40.7 (2.7)	44.3 (5.1)	31.4 (3.3)
AUC_0–120_ glucose, mmol/l × min	1137 (52)	1088 (51)	969 (41)	819 (21)**^,†^
AUC_0–120_ incremental glucose, mmol/l × min	387 (40)	371 (38)	276 (35)	166 (33)**^,††^
Fasting insulin, pmol/l	46.5 (6.3)	51.4 (7.0)	37.5 (6.3)	44.5 (12.5)
Peak insulin, pmol/l	639 (111)	590 (83)	549 (76)	507 (83)
Peak insulin time, min	61.3 (6.4)	65.3 (8.5)	46.4 (6.2)	40.7 (4.0)
AUC_0–120_ insulin, pmol/l × min	45,565 (7881)	42,960 (6048)	37,437 (5652)	31,858 (4879)
AUC_0–120_ incremental insulin, pmol/l × min	39,941 (7296)	36,805 (5366)	32,957 (5036)	26,533 (3571)
AUC_0–120_ glucose/AUC_0–120_ insulin	0.25 (0.04)	0.25 (0.04)	0.22 (0.02)	0.21 (0.02)
Matsuda index	4.93 (0.72)	5.56 (1.16)	5.96 (0.65)	6.63 (0.73)
HOMA-IR	1.93 (0.26)	2.02 (0.28)	1.40 (0.24)	1.71 (0.51)
IGI, pmol/mmol	77 (12)	75 (14)	100 (19)	138 (23)**^,†^
DI_o_	1.69 (0.21)	1.68 (0.34)	2.67 (0.30)	3.84 (0.54)**^,††^

Values are presented as mean (SEM). Data are from all participants that completed the study, apart from *n* = 1 in the sitagliptin group, who was excluded due to pharmacy error

Mixed model analysis was used for statistical comparison

**p < 0.003 for week 0 vs week 12; ^†^0.003 < *p* < 0.05 and ^††^*p* < 0.003 for placebo vs sitagliptin. Bonferroni-corrected level of significance is 0.003 (*α* = 0.05/15)

### Sitagliptin lowers serum triacylglycerol reflected by decreased concentrations of large and medium-sized VLDL particles

**Fig. 2:**
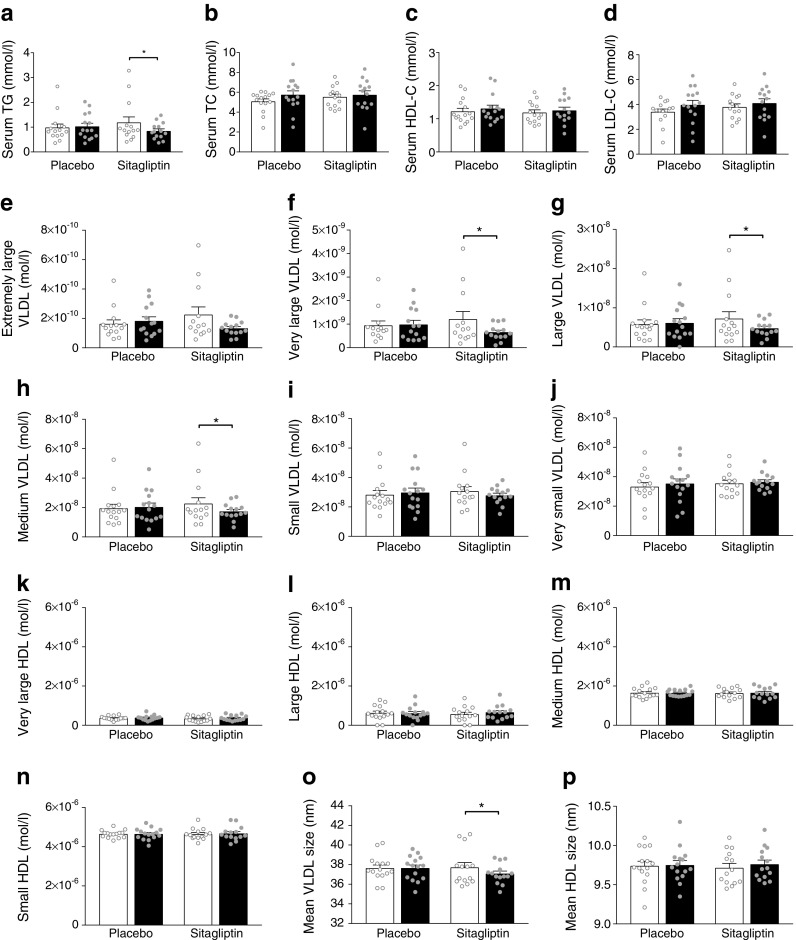
The effect of sitagliptin on serum lipid and VLDL and HDL particle concentration in overweight men with prediabetes. Serum was collected before (week 0, white bars) and after (week 12, black bars) treatment with placebo (*n* = 15) or sitagliptin (*n* = 14). Enzymatic assays were used to measure serum triacylglycerol (TG) (**a**) and total cholesterol (TC) (**b**). HDL-cholesterol was determined by the general hospital laboratory of the LUMC (**c**) and LDL-cholesterol was calculated using the Friedewald equation (**d**). NMR was used to measure serum concentrations of extremely large (**e**), very large (**f**), large (**g**), medium-sized (**h**), small (**i**) and very small (**j**) VLDL particles, and very large (**k**), large (**l**), medium-sized (**m**) and small (**n**) HDL particles. In addition, mean VLDL (**o**) and mean HDL (**p**) particle size was determined. Data are presented as means ± SEM and as individual measurements. Mixed model analysis was used for statistical comparison. *0.003 < *p* < 0.05 for week 0 vs week 12, not significant with Bonferroni-corrected level of significance 0.003 (*α* = 0.05/16)

Previous studies in individuals with type 2 diabetes showed that sitagliptin improves plasma lipid levels [5, 6]. Therefore, we assessed whether sitagliptin also affects serum lipids and lipoprotein levels in overweight individuals with prediabetes. Sitagliptin induced a substantial reduction in serum triacylglycerol levels (−29%; *p* < 0.05 [not significant with a Bonferroni-corrected level of significance of 0.003]; Fig. 2a) without affecting serum total cholesterol (Fig. 2b), total HDL-cholesterol (Fig. 2c), total LDL-cholesterol (Fig. 2d), NEFA levels (data not shown) or plasma catecholamines (data not shown). Placebo did not significantly affect serum triacylglycerol, total cholesterol, HDL-cholesterol or LDL-cholesterol (Fig. 2a–d). The sitagliptin-induced reduction in triacylglycerol was not significantly different from placebo.

Since serum triacylglycerol is carried by lipoproteins, we next assessed whether the reduction in triacylglycerol induced by sitagliptin was specific for certain lipoprotein subfractions. Sitagliptin lowered serum concentrations of very large (−46%; *p* < 0.05; Fig. 2f), large (−35%; *p* < 0.05; Fig. 2g) and medium-sized VLDL particles (−24%; *p* < 0.05; Fig. 2h), although these were all non-significant with a Bonferroni-corrected level of significance of 0.003. Concentrations of small (Fig. 2i) and very small VLDL particles (Fig. 2j) were unchanged. Overall, this resulted in a decrease in mean VLDL particle size (−1.7%; *p* < 0.05 [not significant with a Bonferroni-corrected level of significance of 0.003]; Fig. 2o). In addition, sitagliptin did not significantly change the concentrations of very large, large, medium-sized or small HDL particle subfractions (Fig. 2k–n) or mean HDL particle size (Fig. 2p). Intermediate-dense lipoprotein (IDL), large, medium-sized and small LDL particle concentrations and mean LDL particle size were not affected by sitagliptin treatment (ESM Fig. 2). Placebo did not alter the measured lipoprotein particle concentrations or particle sizes (Fig. 2e–p; ESM Fig. 2). The sitagliptin-induced reduction in concentration of very large, large or medium-sized VLDL particles was not significantly different from placebo, nor was the sitagliptin-induced decrease in VLDL and increase in HDL mean particle size.

In line with these findings, most components of the lipoprotein subclasses in extremely large, very large, large and medium-sized VLDL particles were decreased upon sitagliptin treatment (ESM Table 5). In IDL, only non-esterified cholesterol significantly increased and in HDL particles only phospholipids in very large HDL significantly increased (ESM Table 5), although these were not significant with a Bonferroni-corrected level of significance of 0.0006. Components of the lipoprotein subclasses in LDL fractions (ESM Table 5) and fatty acid composition did not change significantly.

### Sitagliptin does not affect overall REE or substrate preference

To assess whether sitagliptin improves glucose and lipid metabolism by altering substrate utilisation or REE, indirect calorimetry was performed. REE was similar before and after treatment with either sitagliptin or placebo, also when correcting for lean body mass (ESM Table 6). In addition, non-shivering thermogenesis, the respiratory quotient, glucose and lipid oxidation did not change upon treatment (ESM Table 6).

### Sitagliptin increases [^18^F] FDG uptake in subcutaneous WAT, but not in BAT

Next, we assessed whether sitagliptin improves glucose tolerance and lipid profile through beneficial effects on BAT, WAT or skeletal muscle. To this end, we assessed cold-induced [^18^F] FDG uptake in these organs following PET/CT scan. Concerning the primary endpoint, we found that 12 weeks of sitagliptin treatment did not affect [^18^F] FDG uptake in all measured BAT depots, though mean [^18^F] FDG uptake in BAT was very low in most participants (ESM Table 7). Furthermore, sitagliptin did not alter [^18^F] FDG uptake in skeletal muscle or paracolic WAT. Interestingly, [^18^F] FDG uptake in subcutaneous WAT (sWAT) was increased by sitagliptin (+53%; *p* < 0.05 [not significant with Bonferroni-corrected level of significance of 0.002]) but not placebo treatment (ESM Table 7). The sitagliptin-induced effect on [^18^F] FDG uptake in sWAT did not significantly differ from placebo. In the placebo group, [^18^F] FDG uptake by the trapezius muscles was increased (+15%; *p* < 0.05), as was uptake by the descending aorta (+13%; *p* < 0.05), as reference tissues (neither significant with Bonferroni-corrected level of significance of 0.002; ESM Table 7).

### Sitagliptin increases *PGC1β* expression in skeletal muscle

**Fig. 3:**
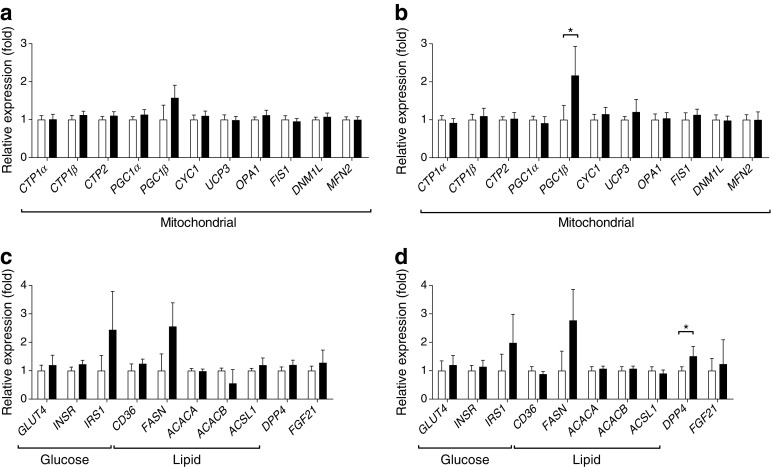
The effect of sitagliptin on skeletal muscle gene expression in overweight men with prediabetes. A fasted skeletal muscle biopsy was taken before (white bars) and after (black bars) 12 weeks of treatment with placebo (*n* = 13) or sitagliptin (*n* = 11). qPCR was used to determine expression of genes involved in mitochondrial function, glucose metabolism and lipid metabolism, as well as *DPP4* and *FGF21*, upon placebo (**a**, **c**) and sitagliptin (**b**, **d**) treatment. Data are presented as means ± SEM. Expression levels were normalised using the mRNA content of the housekeeping gene β-actin (*ACTB*) and expressed as fold change using the ${2}^{-{\Delta \Delta \mathrm{C}}_{\mathrm{t}}}$ method. Mixed model analysis was used for statistical comparison. *0.002 < *p* < 0.05 for week 0 vs week 12, not significant with Bonferroni-corrected level of significance 0.002 (*α* = 0.05/21)

Next, we analysed skeletal muscle biopsies for pathways involved in mitochondrial function, glucose and lipid metabolism. Sitagliptin increased the expression of *PGC1β* (also known as *PPARGC1B*) (+117%; *p* < 0.05 [not significant with Bonferroni-corrected level of significance of 0.002]; Fig. 3b) in skeletal muscle. Sitagliptin also increased *DPP4* gene expression (+51%; *p* < 0.05 [not significant with Bonferroni-corrected level of significance of 0.002]; Fig. 3d). Expression of genes involved in glucose or lipid metabolism were not significantly changed by sitagliptin (Fig. 3d). In the placebo group, expression levels of none of the measured genes were significantly changed upon treatment (Fig. 3a, c).

## Discussion

The aim of the current study was to evaluate the effect of sitagliptin on glucose tolerance, plasma lipids, energy expenditure, skeletal muscle metabolism and [^18^F] FDG uptake by BAT and WAT in overweight men with prediabetes. Here, we report that 12 weeks of sitagliptin treatment improved glucose tolerance and lipid profile in these men. These beneficial effects were accompanied by an increase in mRNA expression of *PGC1β*, a gene encoding an inducer of mitochondrial biogenesis, in skeletal muscle. Sitagliptin did not affect [^18^F] FDG uptake by BAT but increased [^18^F] FDG uptake by sWAT. To our knowledge, this study is the first to provide extensive insight into the beneficial effects of sitagliptin on both glucose and lipid metabolism in overweight individuals with prediabetes, as previous studies mostly focused on individuals with type 2 diabetes [6].

We showed that the improvement in glucose tolerance following treatment of these men with sitagliptin was not explained by changes in body composition. The improved glucose tolerance is in line with previous reports [33, 34]. Since we observed an improvement in IGI (i.e. measure of early insulin secretion) and DI_o_ (i.e. estimation of beta cell function relative to measure of insulin resistance) during OGTT, we speculate that the improved glucose tolerance can be explained by increased early insulin secretion, in turn possibly due to improved beta cell function. In accordance with this, the DPP4 inhibitor vildagliptin (100 mg/day) improved insulin sensitivity and beta cell function in prediabetic individuals upon 6 weeks of treatment [35]. Interestingly, animal studies have shown that sitagliptin can delay the progression of prediabetes into diabetes [36, 37]. Whether this is also the case in humans remains to be elucidated [38].

We also showed that sitagliptin lowered serum triacylglycerol levels in overweight men with prediabetes. This is in line with previous studies showing that sitagliptin reduces triacylglycerol levels in individuals with type 2 diabetes [5, 39], although, in those individuals, total cholesterol levels were also reduced. We used NMR metabolomics to evaluate which triacylglycerol-containing lipoprotein classes were lowered. Thereby, we specifically observed a decrease in the concentration of very large, large and medium-sized VLDL particles, which may point to reduced hepatic VLDL-triacylglycerol synthesis and/or enhanced VLDL-triacylglycerol clearance. Individuals with type 2 diabetes display increased hepatic synthesis of VLDL particles, resulting in elevated VLDL-triacylglycerol [4, 40], presumably due to the impaired ability of insulin to suppress VLDL synthesis. Tremblay et al [6] showed that 6 weeks of sitagliptin treatment reduces triacylglycerol levels due to reduced hepatic VLDL synthesis [6]. Therefore, it is likely that sitagliptin also attenuates hepatic VLDL-triacylglycerol production in our study in prediabetic men who also have impaired insulin sensitivity. A contribution made by the increased lipolysis of VLDL-triacylglycerol by LPL-containing peripheral tissues, including skeletal muscle and BAT, cannot be ruled out, especially since enhanced glucagon-like peptide-1 (GLP-1) receptor signalling has been shown to largely increase the uptake of triacylglycerol-derived fatty acids by BAT in mice [41]. Evaluation of the potential contribution made by accelerated triacylglycerol clearance to the triacylglycerol-lowering effect of sitagliptin should be the subject of future studies.

So far, the contribution of the various metabolic tissues responsible for the beneficial metabolic effects of sitagliptin has not been clarified. We investigated BAT, WAT and skeletal muscle, as those tissues are known to play an important role in energy metabolism. Despite us defining the primary endpoint of this study as the effect of sitagliptin on BAT volume and activity, we did not observe an effect of sitagliptin on [^18^F] FDG uptake by BAT. However, sitagliptin is suggested to activate BAT in rodents, as judged from increased expression of uncoupling protein 1 gene [8]. It should be realised that the read-outs of BAT activity were different in rodents compared with humans. In addition, compared with lean individuals, it is known that [^18^F] FDG uptake in BAT is lower in obese individuals [42], in individuals with type 2 diabetes [43] and in increasing age [44], likely due to increased insulin resistance. Importantly, insulin resistance does not reduce the uptake by BAT of 14(*R*,*S*)-[^18^F]fluoro-6-thia-heptadecanoic acid ([^18^F]FTHA; a fatty acid tracer) or [^11^C] acetate (a measure of oxidative metabolism) [43]. It is feasible that our overweight men with prediabetes are insulin resistant at the level of BAT and, as a consequence, display low [^18^F] FDG uptake in BAT. Therefore, it cannot be ruled out that in our study population [^18^F] FDG uptake by BAT reflects insulin resistance rather than BAT metabolism. Since fatty acids rather than glucose form the main substrate for BAT thermogenesis [45, 46], future studies should preferably use lipid-based PET/CT tracers such as [^18^F] FTHA or PET/CT tracers imaging oxidative capacity, such as [^11^C] acetate, to better reflect BAT activity, especially in insulin-resistant individuals. Interestingly, sitagliptin increased [^18^F] FDG uptake in sWAT, possibly pointing to browning of this tissue. Indeed, enhanced GLP-1 receptor signalling induces massive browning of WAT in mice [41]. Unfortunately, sWAT biopsies were not collected, so it remains unknown whether expression levels of thermogenic genes in WAT are increased upon sitagliptin treatment. Assessment of browning would be important in future human studies using DPP4 inhibitors or GLP1 agonists and would be of particular clinical relevance to overweight participants, who display large subcutaneous WAT depots.

Besides BAT, skeletal muscle is an important contributor to energy metabolism in humans. Sitagliptin increased *DPP4* expression in skeletal muscle, which seems counterintuitive since high local DPP4 levels are thought to impair insulin signalling and thereby induce/deteriorate the development of type 2 diabetes [47]. The increased *DPP4* expression may be a consequence of a compensatory mechanism of increased plasma GLP-1 as a result of DPP4 inhibition, although unfortunately we were not able to measure circulating GLP-1 levels. We did show that sitagliptin increased mRNA expression of *PGC1β*, which is in line with a previous rodent study showing that sitagliptin treatment increases mitochondrial gene expression in skeletal muscle [8]. Since *PGC1β* modulates mitochondrial function through induction of mitochondrial biogenesis [48, 49], sitagliptin may enhance skeletal muscle metabolism via this mechanism. Although we did not detect a significant effect of sitagliptin on energy expenditure, a potential contribution to whole-energy metabolism may not have been picked up by indirect calorimetry. Since skeletal muscles contribute largely to the composition of the human body, a small change in muscle respiration could have a large effect on total body metabolism.

A strength of our study is that we analysed the effect of sitagliptin on the main metabolically active organs. Moreover, we analysed multiple BAT and WAT depots by [^18^F] FDG PET/CT. We also performed an extensive analysis of the serum lipoprotein profile using NMR metabolomics. A limitation of the current study is that we made use of the radiotracer [^18^F] FDG, so may have underestimated the metabolic activity of insulin-resistant tissues in individuals with prediabetes. We performed all measurements after an overnight fast. Since DPP4 inhibitors are most effective after a meal, it might be interesting to investigate the effects of sitagliptin on postprandial glucose and lipid metabolism, which might be even more pronounced. Furthermore, the relatively small sample size might have limited the statistical power. A strength and limitation is that we assessed many variables, which necessitated Bonferroni correction. The Bonferroni threshold for significance was reached only for reduction in glucose excursions by sitagliptin, the lower peak glucose levels during the OGTT and the improvement in the IGI and DI_o_. In addition, we only investigated Europid men. We chose this specific group since South Asians, another large ethnic group within the Netherlands, generally display more insulin resistance and dyslipidaemia (reviewed in [50]) as well as higher GLP-1 levels [51] compared with Europids. Combination of several ethnic groups may have increased variation within our study groups. However, future studies should investigate whether these results also apply to the general population, including women.

In conclusion, we show that 12 weeks of sitagliptin treatment improves glucose tolerance and lipid profile in overweight men with prediabetes. Those effects might be mediated by browning of sWAT and/or increased energy metabolism in skeletal muscle possibly by upregulation of *PGC1β*. However, the precise mechanism linking DPP4 inhibition to metabolic health still remains to be elucidated. Since up to 70% of prediabetic individuals eventually develop type 2 diabetes and current lifestyle and exercise programmes are often difficult to maintain in the long term, further studies on the effect of sitagliptin in preventing or delaying the progression of prediabetes and dyslipidaemia in individuals at risk for developing type 2 diabetes are warranted.

## Electronic supplementary material

ESM (PDF 605 kb)

## Data Availability

The datasets generated during and/or analysed during the current study are available from the corresponding author on reasonable request.

## Abbreviations

BAT: Brown adipose tissue
DI_o_: Oral disposition index
DPP4: Dipeptidyl peptidase-4
[^18^F]FDG: [^18^F]Fluorodeoxyglucose
[^18^F]FTHA: 14(*R*,*S*)-[^18^F]Fluoro-6-thia-heptadecanoic acid
GLP-1: Glucagon-like peptide-1
IDL: Intermediate-dense lipoprotein
IGI: Insulinogenic index
LUMC: Leiden University Medical Center
NMR: Nuclear magnetic resonance
PET/CT: Positron emission tomography/computed tomography
qPCR: Quantitative PCR
REE: Resting energy expenditure
sWAT: Subcutaneous WAT
SUV: Standardised uptake value
WAT: White adipose tissue
