# Supplementary material for: Effect of sitagliptin on energy metabolism and brown adipose tissue in overweight individuals with prediabetes: a randomised placebo-controlled trial
Source: Diabetologia. 2018 Aug 25;61(11):2386–97. doi: 10.1007/s00125-018-4716-x (PMC6182651; doi:10.1007/s00125-018-4716-x)
Supplement: Supplementary file 1 — (PDF 605 kb) [file 125_2018_4716_MOESM1_ESM.pdf]

ELECTRONIC SUPPLEMENTARY MATERIAL

METHODS

Participants

Thirty-two overweight (BMI 25-35 kg/m^2) Dutch Europid men with prediabetes, aged 35-55 years, were included in the study. Participants were recruited via local advertisements and underwent medical screening prior to participation, including an interview regarding medical history, physical examination, a blood chemistry test and an oral glucose tolerance test (OGTT) to exclude individuals with undiagnosed type 2 diabetes. Prediabetes was defined as having a fasted serum glucose between 5.6 mmol/l and 6.9 mmol/l, according to the American Diabetes Association (ADA) criteria [1], and/or a plasma glucose level between 7.8 mmol/l and 11.1 mmol/l following an OGTT, according to World Health Organization (WHO) criteria for impaired glucose tolerance. Exclusion criteria were type 2 diabetes (*e.g.* fasted glucose > 6.9 mmol/l and/or plasma glucose following OGTT > 11.1 mmol/l), smoking, recent weight change (> 3 kg within the last 3 months), rigorous exercise, use of beta-blockers, uncontrolled hypertension, hyper- or hypothyroidism, renal failure or liver dysfunction, or a positron emission tomography/computed tomography (PET/CT) scan within the last year. The study was approved by the Medical Ethical Committee of the Leiden University Medical Center and performed in accordance with the principles of the revised Declaration of Helsinki. Written informed consent was obtained from all volunteers prior to participation. Thirty participants were initially randomised. Two participants withdrew from the study prior to completion (both in the sitagliptin group; one participant because of heartburn and one participant because of joint pain) and were subsequently replaced, with two new participants, that were allocated to the same treatment. All symptoms resolved

without sequelae. Thus, thirty participants completed the study. Also, one participant from the sitagliptin group was excluded from analysis, due to a distribution error where the participant received both sitagliptin and placebo as treatment.

Study design

Participants were enrolled in a randomised, double-blinded, placebo-controlled study that was conducted in the Leiden University Medical Center (Leiden, The Netherlands) and Alrijne Hospital (Leiderdorp, The Netherlands). The primary study endpoint was the effect of sitagliptin treatment on brown adipose tissue volume and activity in overweight men with prediabetes. Secondary endpoints were the effect of sitagliptin on body weight and body composition, resting energy expenditure, fasting markers in blood for glucose and lipid metabolism, [^{18}F]FDG uptake by white adipose tissue and skeletal muscle and expression of genes involved in insulin signalling, glucose and lipid metabolism in skeletal muscle biopsies. As a post-hoc analysis we quantified lipid and lipoprotein composition using high-throughput proton NMR metabolomics. Participants were randomly assigned in a 1:1 ratio by the unblinded hospital pharmacist to receive oral administration of either sitagliptin (Sitagliptin phosphate, Januvia, Merck Sharp and Dome, Haarlem, The Netherlands) at a dose of 100 mg oral daily, or placebo for 12 weeks. Following initial screening, participants were studied at baseline and after 12 weeks of treatment. Before and after the treatment period there were two measurement days. During the first day, which took place at the Alrijne hospital in Leiderdorp, body composition (DEXA, iDXA, GE Healthcare, Little Chalfont, UK), skin temperature (iButton, Maxim Integrated Products, San Jose, CA, USA), thermoneutral and cold-exposed resting energy expenditure (REE; indirect calorimetry, Oxycon Pro, CareFusion, Heidelberg, Germany), and cold-induced [^{18}F]fluorodeoxyglucose ([^{18}F]FDG) uptake by BAT and other metabolic organs such as white adipose tissue (WAT)

and skeletal muscle were determined by PET/CT scan (Gemini TF-64, Philips Healthcare, Best, The Netherlands). In addition, thermoneutral and cold-exposed venous blood samples were collected. On the second day at the Leiden University Medical Center, a fasting skeletal muscle biopsy was taken from the vastus lateralis muscle followed by an OGTT. All measurements took place after participants had fasted for 10h overnight and had consumed a standardised dinner the night before. In addition, participants were asked to refrain from caffeine and alcohol intake or strenuous physical activity 24 h prior to the study days. Each week during the treatment period, participants measured their blood glucose and were contacted by the investigator to monitor compliance, adverse events or signs of hypoglycemia. In addition, participants were instructed not to alter their lifestyle during the study period. The study was conducted between March 2015 and September 2016.

Indirect calorimetry, individualised cooling protocol and [^{18}F]FDG PET-CT scan

On the first measurement day an intravenous cannula was placed in antecubital vein for blood sampling during thermoneutral and mild cold conditions and injection of the [^{18}F]FDG tracer. First detailed body composition was obtained by dual-energy X-ray absorptiometry, followed by placement of 14 wireless temperature sensors at 14 prescribed ISO-defined places [2]. Mean skin temperature was calculated as the average of all iButtons. Distal skin temperature was calculated as the average temperature of the hand and feet. Proximal skin temperature was defined as the average of the iButtons on the chest, abdomen, scapula and lower back. Next participants were placed in a bed in semi-supine position between two water-perfused mattresses (BlanketRol® III Sub-Zero (CSZ) Products, Cincinnati, OH, USA). As described previously [3], the protocol started with 1 h of thermoneutrality (water temperature 32°C) at which thermoneutral REE was measured for 30 minutes using indirect calorimetry and a venous blood sample was drawn. After1 h at thermoneutrality, participants were

progressively cooled until their shivering point or until they reached the minimum water temperature of 9°C. At this mild cold condition REE was measured for another 30 minutes and a second blood sample was collected. Oxygen consumption and carbon dioxide production were determined every minute. REE and respiratory quotient (RQ) were calculated and substrate utilization was assessed by calculation of lipid and glucose oxidation after correction for protein oxidation as described earlier [4]. Non-shivering thermogenesis (NST) was assessed by the difference in REE during mild cooling compared to thermoneutral conditions. Subsequently, 110 MBq of [^{18}F]FDG was injected intravenously and after 1 h of incubation, the PET/CT imaging protocol started with a low-dose CT scan (120 kV, 30 mAs), immediately followed by a PET scan (10 bed positions, 4 minutes per bed position) acquired according to EARL standards [5] to assess [^{18}F]FDG uptake by BAT, WAT and skeletal muscle from skull to pelvis. In the sitagliptin group one participant became claustrophobic inside the PET/CT scan and could therefore not finish this measurement.

Skeletal muscle biopsy and oral glucose tolerance test

On the second measurement day, a fasted skeletal muscle biopsy was taken from the vastus lateralis muscle according to the technique of Bergström [6]. Subsequently, the samples were frozen in liquid nitrogen and stored at -80° C until further analysis. After 1 h of rest, a glucose tolerance was assessed using a 75-g OGTT. A cannula was inserted in the antecubital vein for blood sampling and samples were drawn at respectively t = -10, 0, 10, 20, 30, 40, 50, 60, 90, 120 minutes after ingestion of the glucose drink. Serum was obtained, snap-frozen in liquid nitrogen and stored at -80°C until further analysis.

Analysis

Serum measurements

Commercially available enzymatic kits were used to measure serum concentrations of triacylglycerol, total cholesterol (Roche Diagnostics, Woerden, The Netherlands), NEFA (Wako Chemicals, Neuss, Germany) and glucose (Instruchemie, Delfzijl, The Netherlands). Insulin concentrations were measured using ELISA (Crystal Chem Inc., Elk Grove Village, IL, USA). The intra-assay coefficients of variability (CV) were 3.8%, 4.2%, 3.6%, 2.5% and 5.9% for triacylglycerol, total cholesterol, free fatty acids, glucose and insulin respectively. The inter-assay CVs were 5.3%, 4.0%, 7.9%, 4.7% and 7.1% for triacylglycerol, total cholesterol, free fatty acids, glucose and insulin respectively. Plasma catecholamines were measured in the laboratory of Vascular Medicine (Erasmus MC, Rotterdam, The Netherlands) using standard procedure. Aspartate aminotransferase, alanine aminotransferase, γ-glutamyltransferase, HbA1c and HDL-cholesterol were determined by the general hospital Laboratory of the Leiden University Medical Center and LDL-cholesterol was calculated using the Friedewald formula (LDL; *Total Cholesterol-HDL-(Triacylglycerol/2.17))* [8]. In addition, lipid and lipoprotein composition was quantified using high-throughput proton NMR metabolomics (Nightingale Health, Helsinki, Finland). This method provides simultaneous quantification of routine lipids, lipoprotein subclass profiling with lipid concentrations within 14 subclasses, fatty acid composition, and various low-molecular metabolites including amino acids, ketone bodies and gluconeogenesis-related metabolites in molar concentration units. The following components of the lipoprotein subclasses were quantified: phospholipids, triacylglycerol, total cholesterol, non-estrified cholesterol, and cholesteryl esters. The mean size for VLDL, LDL and HDL particles was calculated by weighting the corresponding subclass diameters with their particle concentrations [9]. Details of the experimentation and applications of the NMR metabolomics platform have been described previously [10]. Data were analysed using SoftMaxPro 5.4.1 software. For the analysis of the OGTT, theAUC was calculated using the trapezoidal rule [11]. Incremental

AUC was calculated by deducting the area below the baseline value from total AUCs. Insulin sensitivity was estimated using the Matsuda index [12]. The insulinogenic index (IGI; $\Delta I_{0-30}/\Delta G_{0-30}$, where I is insulin and G is glucose) was used as a measure of early insulin secretion [13]. The oral disposition index (DI_0; [$\Delta I_{0-30}/\Delta G_{0-30}$]/fasting insulin) was used to estimate beta cell function relative to the prevailing level of insulin resistance [14].

PET/CT scan analysis

[^{18}F]FDG uptake by BAT, WAT and skeletal muscle was determined from the [^{18}F]FDG PET/CT scan using Fiji ImageJ 1.51d (Beth Israel Deaconess Medical Center, Beth, Israel) [7] and analysed by two researchers (KJN, BMT) blinded to allocation. In the region of interest (ROI), the bilateral cervical and clavicular regions, mediastinal and paravertebral BAT areas were autocontoured using an set personalised standardised uptake value (SUVindiv) threshold with a tissue radiodensity between -190 and -10 Hounsfield units [15]. SUVindiv threshold was calculated with the following formula: 1.2/lean body mass (in kg)/body mass (in kg), according to the latest expert panel recommendations [16]. BAT metabolic volume (BMV) was measured in millilitres, BAT activity was reported in terms of SUV (the ratio of activity in kBq/mL within the ROI and the injected activity [kBq] per bodyweight [g]). Both SUV for the hottest single voxel (SUVmax) and mean SUV for all voxels within a BAT region (SUVmean) are reported. For WAT areas (subcutaneous and paracolic), skeletal muscles (sternocleidomastoid, longus colli, trapezius, deltoid, pectoralis major, psoas major, and gluteus maximus muscle) and reference tissues (liver, cerebellum and descending aorta), an SUV threshold was set at 0 and no Hounsfield units threshold was applied.

qPCR analysis in skeletal muscle biopsies

Skeletal muscle biopsies were analysed for expression of genes involved in insulin signalling (*INSR, IRS1)*, glucose metabolism (*GLUT4*), lipid metabolism (*ACACA*, *ACACB*, *ACSL1, CD36, FASN)*, and mitochondrial function (*CTP1α, CTP1β, CTP2, CYCS, DNM1L, MFN2, OPA1, PPARGC1α* [also known as *PPARGC1A*], *PPARGC1β* [also known as *PPARGC1B*],, *UCP3)*, as well as DPP4 (*DPP4)* and Fibroblast Growth Factor (*FGF21)* using qPCR (Bio-Rad CFX96; Veenendaal, The Netherlands) (see ESM Table 1 for primer sequences). Bio-Rad CFX Manager software version 3.1 (Bio-Rad Laboratories Inc, Hercules, CA, USA) was used for analysis and quantification. Biopsy material of five participants (two from placebo and three from sitagliptin group) was insufficient for reliable mRNA analysis. Expression levels were normalised using the mRNA content of the housekeeping gene β -Actin (*ACTB*) and expressed as fold change using the $2^{-\Delta\Delta C_t}$ method.

Statistical analysis

Power calculations were made for the primary outcome measurement of BAT activity (SUVmean). On the basis of previous studies [17], we anticipated a 20% increase in BAT activity after Sitagliptin treatment. An SUVmean of 2.20 was expected in the control group and a SUVmean of 2.64 in the sitagliptin group after intervention, with a SD of 0.42. Assuming a bilateral alternative, we were able to detect differences of at least 20% in SUVmean with a power of more than 80% and an alpha of 0.05 in a group of 30 participants. Data was analysed using SPSS Statistics (version 23.0; IBM Corporation, Armonk, NY, USA). Data are shown as mean ± S.E.M, unless stated otherwise. Two-tailed unpaired Student's *t*-test was used to compare baseline characteristic between sitagliptin and placebo group. Mixed model analyses with treatment and occasion as fixed effects and subject specific deviances from the mean as random effects were used to assess the effect of the

treatment. Ifthe mixed model failed to converge a non-parametric paired test (Wilcoxon Signed-Rank Test) was used. Statistical results are shown with adjustment for multiple testing. Bonferroni corrected levels of significance are shown in the table and figure legends.

TABLES AND FIGURES

ESM Table 1. Primer sequences of forward and reverse primers for qRT-PCR

Gene	Forward primer	Reverse primer
ACACA	Ordered by Qiagen	Ordered by Qiagen
ACACB	Ordered by Qiagen	Ordered by Qiagen
ACSL1	Ordered by Qiagen	Ordered by Qiagen
ACTB	Ordered by Qiagen	Ordered by Qiagen
CD36	Ordered by Qiagen	Ordered by Qiagen
CTP1α	CCAGACGAAGAACGTGGTCA	ATCTTGCCGTGCTCAGTGAA
CTP1β	GGATGTTCAACACCACTCGG	TCTCCAGATCCTGAGGCTTGA
CTP2	CAGGCTGCCTATTCCCAAACT	CCAGGGTCCCGAAATGTAGC
CYCS	CACTGCGGGAAGGTCTCTAC	TCAACATCTTGAGCCCCATGC
DNM1L	GTTGATCCACTTGGTGGCCT	CATGAACCAGTTCCACACAGC
DPP4	GCAGAATGTCCAGATGCCCT	GTGCTTGCAAGGTAAGTGGC
FASN	CTCCTTCTTCGGAGTCCACC	AGTGTGTGTTCCTCGGAGTG
FGF21	GCAGCGGTACCTCTACACAG	GCACAGGAACCTGGATGTCT
FIS1	GTAAAGGCATCGTGCTGCTC	ACGGCCAGGTAGAAGACGTA
GLUT4	GGCTGGAGTCCTGCTTCTGCAC	GCTGGTACATTTGAATCTGCAGCGA
INSR	GGGCAACGGCTCTTGGACGG	CGGCCCATCTGGCTGCCTCTT
IRS1	TGTTTTTCGGAGCCTCCCTC	CCGCCACTTCTTCTCGTTCT
MFN2	GTGGCCCAACTCTAAGTGCC	CCAGCGGTTGTTCAGGATGA
OPA1	TGGACTACAGAGGATGGTGC	TGCCAGGTCTACTTTGGTCA
PPARGC1α	AGTGGTGCAGTGACCAATCA	CTGCTAGCAAGTTTGCCTCA
PPARGC1β	AGCTTTGAGCAGACCTTGACA	TCGCTCTGGGTGCTTCTTTG
UCP3	GAAGGTCCGATTTCAGGCCA	GTAGGTCACCACCTCAGCAC

ESM Table 2. Effect of sitagliptin treatment on body composition in overweight men with prediabetes

	**Placebo (n=15)**		**Sitagliptin (n=14)**	
	Week 0	Week 12	Week 0	Week 12
Total body mass (kg)	93.7 (1.6)	92.7 (1.4)	95.4 (3.1)	94.6 (2.9)
Fat mass (%)	30.1 (1.4)	29.6 (1.6)	29.7 (1.4)	29.4 (1.4)
Lean mass (%)	67.4 (1.3)	67.9 (1.5)	67.7 (1.4)	68.0 (1.4)
Bone mineral density (g/cm^3)	1.32 (0.03)	1.33 (0.03)	1.42 (0.04)	1.42 (0.04)

DEXA scanning was used to measure body composition at baseline (Week 0) and after 12 weeks (Week 12) of placebo (left, *n*=15) or sitagliptin (right, *n*=14) treatment. Data are presented as mean (standard error of the mean). Mixed model analysis was used for statistical comparison. Bonferroni corrected level of significance is 0.01 (alpha = 0.05 / 4).

ESM Table 3. Effect of sitagliptin treatment on skin temperature in overweight men with prediabetes

	**Placebo (*n*=15)**		**Sitagliptin (*n*=14)**	
	Week 0	Week 12	Week 0	Week 12
Delta mean skin temperature TN *vs* COLD (°C)	-5.5 (0.3)	-5.2 (0.3)	-5.6 (0.4)	-5.4 (0.3)
Delta proximal skin temperature TN *vs* COLD (°C)	-7.8 (0.6)	-7.6 (0.7)	-8.0 (0.8)	-7.5 (0.5)
Delta distal skin temperature TN *vs* COLD (°C)	-7.2 (0.7)	-6.3 (0.6)	-6.8 (0.7)	-6.0 (0.5)
Delta supraclavicular skin temperature TN *vs* COLD (°C)	-0.1 (0.2)	0.1 (0.1)	-0.1 (0.2)	-0.3 (0.4)

Data are presented as mean (standard error of the mean). TN; thermoneutral, before cooling. COLD; during mild cooling. Mixed model analysis was used for statistical comparison. Bonferroni corrected level of significance is 0.01 (alpha = 0.05 / 4).

ESM Table 4. Effect of sitagliptin treatment on liver enzymes

	**Placebo (*n*=15)**		**Sitagliptin (*n*=14)**	
	Week 0	Week 12	Week 0	Week 12
Aspartate aminotransferase (µkat/l)	0.52 (0.03)	0.53 (0.03)	0.59 (0.11)	0.47 (0.04)
Alanine aminotransferase (µkat/l)	0.33 (0.03)	0.60 (0.08) **	0.38 (0.08)	0.48 (0.10)
γ-glutamyltransferase (µkat/l)	0.52 (0.06)	0.57 (0.08)	0.51 (0.08)	0.55 (0.09)

Data are presented as mean (standard error of the mean). Mixed model analysis was used for statistical comparison. **$p < 0.01$ week 0 *vs* week 12, significant with Bonferroni corrected level of significance 0.01 (alpha = 0.05 / 3).

ESM Table 5. Effect of sitagliptin on lipoprotein composition

	**Placebo (n=15)**		**Sitagliptin (n=14)**	
	Percentage	*p*-value	Percentage	*p*-value
**Extremely large VLDL particles**				
Composition				
Total Lipids	+12%	ns	-42%	<0.05
Phospholipids	+14%	ns	-47%	<0.05
Total cholesterol	+15%	ns	-48%	<0.05
Cholesterol esters	+17%	ns	-43%	ns
Free cholesterol	+13%	ns	-54%	<0.05
Triacylglycerol	+11%	ns	-34%	<0.05
**Very large VLDL particles**				
Composition				
Total Lipids	+4%	ns	-46%	<0.05
Phospholipids	+6%	ns	-49%	<0.05
Total cholesterol	+6%	ns	-49%	<0.05
Cholesterol esters	+5%	ns	-46%	<0.05
Free cholesterol	+7%	ns	-53%	<0.05
Triacylglycerol	+3%	ns	-45%	<0.05
**Large VLDL particles**				
Composition				
Total Lipids	+14%	ns	-35%	<0.05
Phospholipids	+15%	ns	-35%	<0.05
Total cholesterol	+16%	ns	-35%	ns
Cholesterol esters	+15%	ns	-30%	ns
Free cholesterol	+17%	ns	-41%	<0.05
Triacylglycerol	+12%	ns	-34%	<0.05
**Medium VLDL particles**				
Composition				
Total Lipids	+4%	ns	-23%	<0.05
Phospholipids	+5%	ns	-22%	<0.05
Total cholesterol	+7%	ns	-20%	ns
Cholesterol esters	+8%	ns	-16%	ns
Free cholesterol	+6%	ns	-26%	<0.05
Triacylglycerol	+4%	ns	-25%	<0.05
**Small VLDL particles**				
Composition				
Total Lipids	+6%	ns	-9%	ns
Phospholipids	+6%	ns	-7%	ns
Total cholesterol	+7%	ns	-2%	ns
Cholesterol esters	+8%	ns	+1%	ns
Free cholesterol	+6%	ns	-7%	ns
Triacylglycerol	+4%	ns	-15%	ns
**Extra small VLDL particles**				
Composition				
Total Lipids	+6%	ns	+4%	ns
Phospholipids	+6%	ns	+5%	ns

Total cholesterol	+7%	ns	+7%	ns
Cholesterol esters	+7%	ns	+6%	ns
Free cholesterol	+6%	ns	+7%	ns
Triacylglycerol	+5%	ns	-5%	ns
**IDL particles**				
Composition				
Total Lipids	+5%	ns	+4%	ns
Phospholipids	+4%	ns	+5%	ns
Total cholesterol	+5%	ns	+6%	ns
Cholesterol esters	+5%	ns	+3%	ns
Free cholesterol	+4%	ns	+9%	<0.05
Triacylglycerol	+6%	ns	+2%	ns
**Large LDL particles**				
Composition				
Total Lipids	+4%	ns	+5%	ns
Phospholipids	+4%	ns	+4%	ns
Total cholesterol	+4%	ns	+5%	ns
Cholesterol esters	+5%	ns	+4%	ns
Free cholesterol	+4%	ns	+7%	ns
Triacylglycerol	+7%	ns	+4%	ns
**Medium LDL particles**				
Composition				
Total Lipids	+4%	ns	+4%	ns
Phospholipids	+4%	ns	+2%	ns
Total cholesterol	+4%	ns	+5%	ns
Cholesterol esters	+4%	ns	+6%	ns
Free cholesterol	+3%	ns	+4%	ns
Triacylglycerol	+6%	ns	+4%	ns
**Small LDL particles**				
Composition				
Total Lipids	+4%	ns	+4%	ns
Phospholipids	+3%	ns	+1%	ns
Total cholesterol	+4%	ns	+6%	ns
Cholesterol esters	+4%	ns	+7%	ns
Free cholesterol	+3%	ns	+5%	ns
Triacylglycerol	+6%	ns	-5%	ns
**Very large HDL particles**				
Composition				
Total Lipids	+3%	ns	+9%	ns
Phospholipids	+1%	ns	+14%	<0.05
Total cholesterol	+4%	ns	+7%	ns
Cholesterol esters	+5%	ns	+6%	ns
Free cholesterol	+4%	ns	+9%	ns
Triacylglycerol	+12%	ns	-22%	ns
**Large HDL particles**				
Composition				
Total Lipids	-10%	ns	+9%	ns
Phospholipids	-9%	ns	+8%	ns

Total cholesterol	-11%	ns	+10%	ns
Cholesterol esters	-10%	ns	+9%	ns
Free cholesterol	-10%	ns	+10%	ns
Triacylglycerol	-7%	ns	+15%	ns
**Medium HDL particles**				
Composition				
Total Lipids	-1%	ns	+2%	ns
Phospholipids	+1%	ns	+2%	ns
Total cholesterol	-1%	ns	+2%	ns
Cholesterol esters	-2%	ns	+1%	ns
Free cholesterol	-2%	ns	+7%	ns
Triacylglycerol	+5%	ns	+7%	ns
**Small HDL particles**				
Composition				
Total Lipids	-1%	ns	+1%	ns
Phospholipids	-1%	ns	-1%	ns
Total cholesterol	-1%	ns	+5%	ns
Cholesterol esters	-1%	ns	+6%	ns
Free cholesterol	-1%	ns	-1%	ns
Triacylglycerol	+3%	ns	-8%	ns

NMR was used to determine lipoprotein composition. Values are presented as percent increase (+) or decrease (-) from baseline (week 0). ns: not significant. Mixed model analysis was used for statistical comparison. Bonferroni corrected level of significance is 0.0006 (alpha = 0.05 / 84).

ESM Table 6. Effect of sitagliptin on energy expenditure and substrate utilisation

	**Placebo (*n*=15)**		**Sitagliptin (*n*=14)**	
	Week 0	Week 12	Week 0	Week 12
REE (kJ/day)	4736 (138)	4858 (146)	4807 (163)	4845 (188)
REE corrected for LBM (kJ/day/kg LBM)	75.3 (1.7)	77.4 (1.3)	77.8 (1.3)	75.7 (2.1)
RQ	0.84 (0.01)	0.84 (0.01)	0.84 (0.01)	0.81 (0.02)
Lipid oxidation (g/min)	0.065 (0.005)	0.067 (0.006)	0.069 (0.018)	0.075 (0.005)
Glucose oxidation (g/min)	0.116 (0.004)	0.119 (0.004)	0.122 (0.004)	0.118 (0.006)
Non-shivering thermogenesis (%)	13 (5)	15 (5)	8 (2)	10 (4)

Data are presented as mean (standard error of the mean). Respiratory quotient (RQ) was calculated as vCO_2/vO_2. LBM: lean body mass, NST; non-shivering thermogenesis, REE: resting energy expenditure. Mixed model analysis was used for statistical comparison. Bonferroni corrected level of significance is 0.008 (alpha = 0.05 / 6).

ESM Table 7. Effect of sitagliptin on [^{18}F]FDG uptake in brown and white adipose tissue and skeletal muscle

	Placebo (n=15)		Sitagliptin (n=13)	
	Week 0	Week 12	Week 0	Week 12
**Brown adipose tissue depots**				
Classical BAT depots (cervical + supraclavicular)				
Classical BMV (mL)	22.16 (8.75)	13.88 (4.67)	36.82 (13.17)	31.95 (13.09)
Classical BAT activity (SUV_{mean})	2.25 (0.11)	2.15 (0.08)	2.33 (0.14)	2.27 (0.12)
Classical BAT activity (SUV_{max})	3.94 (0.68)	2.60 (0.40)	4.78 (0.72)	4.43 (0.70)
Total body BAT (cervical + supraclavicular + mediastinal + paravertebral)				
Total BMV (mL)	32.85 (11.35)	25.11 (5.84)	54.25 (16.13)	47.47 (15.85)
Total BAT activity (SUV_{mean})	2.25 (0.09)	2.23 (0.08)	2.31 (0.13)	2.31 (0.12)
Total BAT activity (SUV_{max})	4.41 (0.66)	3.93 (0.38)	5.05 (0.67)	4.82 (0.62)
**White adipose tissue depots (WAT)**				
Subcutaneous (SUV_{mean})	0.22 (0.03)	0.26 (0.03)	0.19 (0.03)	0.29 (0.03) *
Paracolic (SUV_{mean})	0.53 (0.09)	0.59 (0.08)	0.59 (0.08)	0.58 (0.08)
**Skeletal muscles**				
m. Sternocleidomastoideus (SUV_{mean})	1.60 (0.20)	1.64 (0.15)	1.69 (0.33)	1.46 (0.15)
m. Longius colli (SUV_{mean})	2.20 (0.23)	2.63 (0.30)	2.33 (0.12)	2.42 (0.26)
m. Trapezius (SUV_{mean})	0.61 (0.05)	0.70 (0.03) *	0.67 (0.03)	0.67 (0.04)
m. Deltoideus (SUV_{mean})	0.55 (0.02)	0.61 (0.02)	0.63 (0.03)	0.61 (0.02)
m. Pectoralis major (SUV_{mean})	0.84 (0.05)	1.00 (0.08)	0.97 (0.18)	0.93 (0.08)
m. Psoas major (SUV_{mean})	0.96 (0.14)	1.04 (0.17)	0.76 (0.09)	0.91 (0.05)
m. Gluteus maximus (SUV_{mean})	0.56 (0.04)	0.56 (0.02)	0.56 (0.03)	0.60 (0.03)
**Reference tissues**				
Liver (SUV_{mean})	2.56 (0.12)	2.74 (0.08)	2.65 (0.10)	2.73 (0.09)
Cerebellum (SUV_{mean})	7.68 (0.50)	7.97 (0.36)	7.83 (0.39)	7.92 (0.31)
Descending aorta (SUV_{mean})	1.63 (0.09)	1.84 (0.07) *	1.75 (0.05)	1.78 (0.07)

Values are presented as mean (standard error of the mean). BAT: brown adipose tissue, BMV: BAT metabolic volume, SUV: standardised uptake value, WAT: white adipose tissue. Mixed model analysis was used for statistical comparison. * $0.002 < p < 0.05$ week 0 *vs* week 12, not significant with Bonferroni corrected level of significance0.002 (alpha = 0.05 / 18). In the Sitagliptin group one subject was claustrophobic and did not completed the PET/CT scan.

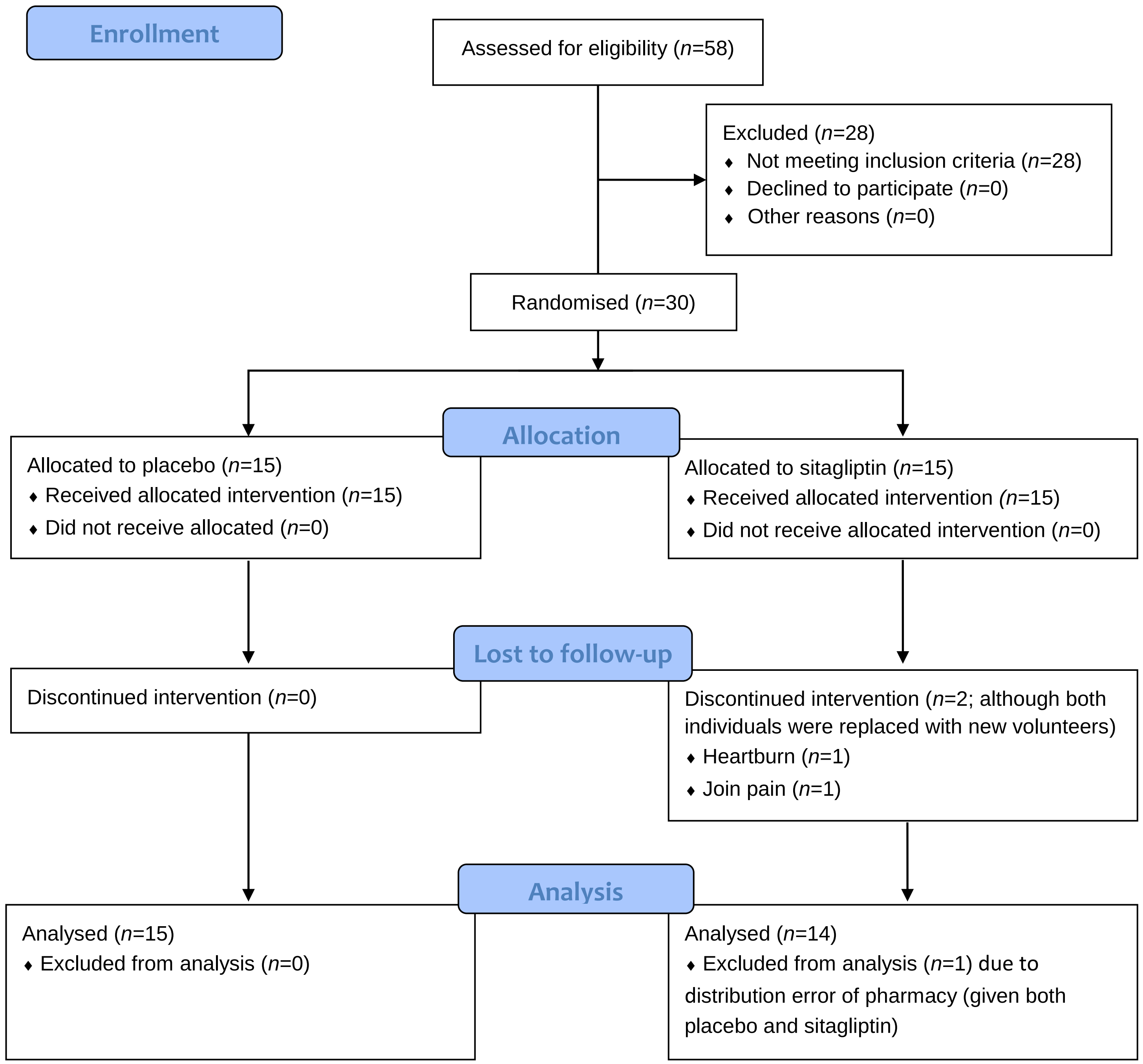

ESM Figure 1. CONSORT flow diagram of the study

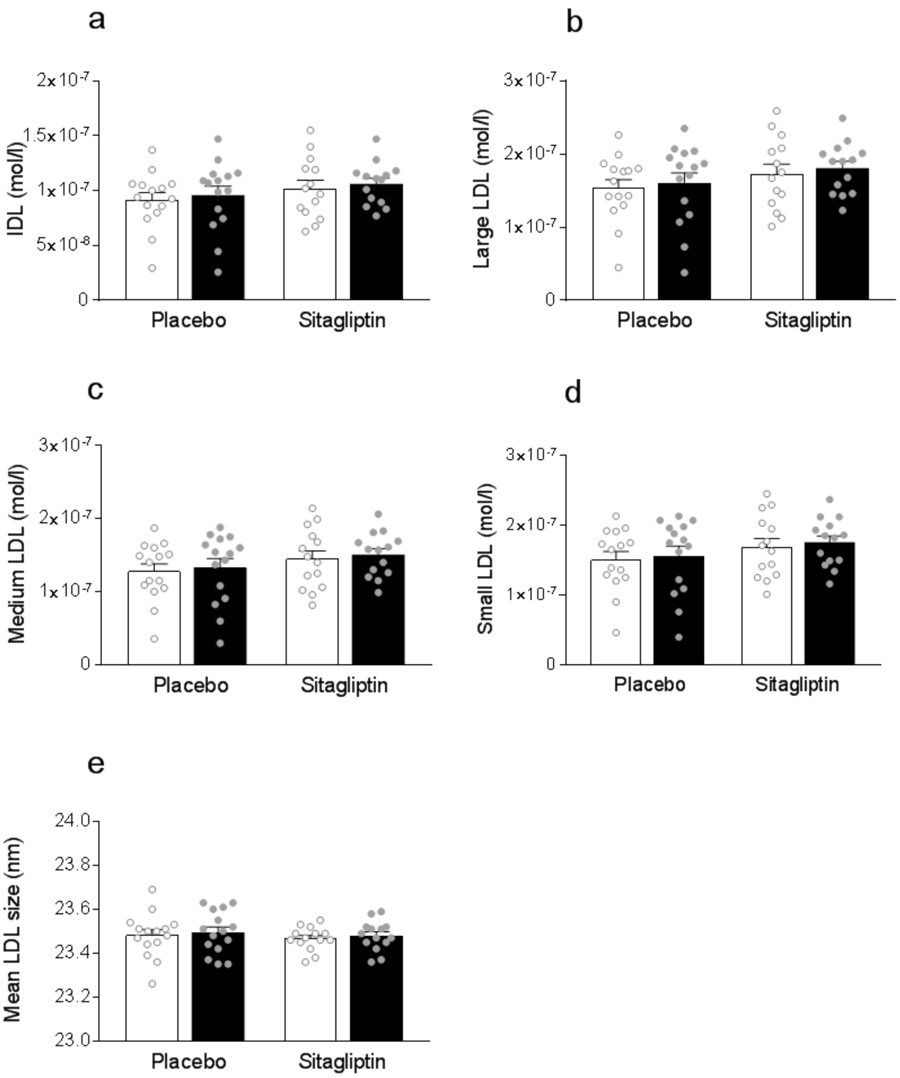

ESM Figure 2 The effect of sitagliptin on serum IDL and LDL particle concentration in overweight men with prediabetes. Serum was collected before (open circles / white bars/ Week 0) and after (closed circles / black bars/ Week 12) 12 weeks of treatment with placebo (*n*=15) or sitagliptin (*n*=14). NMR was used to measure serum IDL (**a**), large- (**b**), medium- **(c)** and small-sized **(d)** LDL particle concentration. In addition, mean LDL particle size (**e**) was determined. Data are presented as mean ± S.E.M., and as individual measurements. Mixed model analysis was used for statistical comparison. Bonferroni corrected level of significance is 0.01 (alpha = 0.05 / 5).

REFERENCES

[1] Chamberlain JJ, Rhinehart AS, Shaefer CF, Jr., Neuman A (2016) Diagnosis and Management of Diabetes: Synopsis of the 2016 American Diabetes Association Standards of Medical Care in Diabetes. Ann Intern Med 164: 542-552

[2] van Marken Lichtenbelt WD, Daanen HA, Wouters L, et al. (2006) Evaluation of wireless determination of skin temperature using iButtons. Physiol Behav 88: 489-497

[3] Bakker LE, Boon MR, van der Linden RA, et al. (2014) Brown adipose tissue volume in healthy lean south Asian adults compared with white Caucasians: a prospective, case-controlled observational study. Lancet Diabetes Endocrinol 2: 210-217

[4] Simonson DC, DeFronzo RA (1990) Indirect calorimetry: methodological and interpretative problems. Am J Physiol 258: E399-412

[5] Boellaard R, Delgado-Bolton R, Oyen WJ, et al. (2015) FDG PET/CT: EANM procedure guidelines for tumour imaging: version 2.0. Eur J Nucl Med Mol Imaging 42: 328-354

[6] Shanely RA, Zwetsloot KA, Triplett NT, Meaney MP, Farris GE, Nieman DC (2014) Human skeletal muscle biopsy procedures using the modified Bergstrom technique. J Vis Exp: 51812

[7] Kanoun S, Tal I, Berriolo-Riedinger A, et al. (2015) Influence of Software Tool and Methodological Aspects of Total Metabolic Tumor Volume Calculation on Baseline [18F]FDG PET to Predict Survival in Hodgkin Lymphoma. PLoS One 10: e0140830

[8] Friedewald WT, Levy RI, Fredrickson DS (1972) Estimation of the concentration of low-density lipoprotein cholesterol in plasma, without use of the preparative ultracentrifuge. Clin Chem 18: 499-502

[9 Kettunen J, Demirkan A, Wurtz P, et al. (2016) Genome-wide study for circulating metabolites identifies 62 loci and reveals novel systemic effects of LPA. Nat Commun 7: 11122

[10] Soininen P, Kangas AJ, Wurtz P, Suna T, Ala-Korpela M (2015) Quantitative serum nuclear magnetic resonance metabolomics in cardiovascular epidemiology and genetics. Circ Cardiovasc Genet 8: 192-206

[11] Matthews JN, Altman DG, Campbell MJ, Royston P (1990) Analysis of serial measurements in medical research. BMJ 300: 230-235

[12] Matsuda M, DeFronzo RA (1999) Insulin sensitivity indices obtained from oral glucose tolerance testing: comparison with the euglycemic insulin clamp. Diabetes Care 22: 1462-1470

[13] Tura A, Kautzky-Willer A, Pacini G (2006) Insulinogenic indices from insulin and C-peptide: comparison of beta-cell function from OGTT and IVGTT. Diabetes Res Clin Pract 72: 298-301

[14] Retnakaran R, Qi Y, Goran MI, Hamilton JK (2009) Evaluation of proposed oral disposition index measures in relation to the actual disposition index. Diabet Med 26: 1198-1203

[15] Martinez-Tellez B, Nahon KJ, Sanchez-Delgado G, et al. (2018) The impact of using BARCIST 1.0 criteria on quantification of BAT volume and activity in three independent cohorts of adults. Sci Rep 8: 8567

[16] Chen KY, Cypess AM, Laughlin MR, et al. (2016) Brown Adipose Reporting Criteria in Imaging STudies (BARCIST 1.0): Recommendations for Standardized FDG-PET/CT Experiments in Humans. Cell Metab 24: 210-222

[17] Hanssen MJ, van der Lans AA, Brans B, et al. (2016) Short-term Cold Acclimation Recruits Brown Adipose Tissue in Obese Humans. Diabetes 65: 1179-1189
